# Lack of detection of SARS-CoV-2 in British wildlife 2020–21 and first description of a stoat (*Mustela erminea*) *Minacovirus*

**DOI:** 10.1099/jgv.0.001917

**Published:** 2023-12-07

**Authors:** Ternenge Apaa, Amy J. Withers, Laura Mackenzie, Ceri Staley, Nicola Dessi, Adam Blanchard, Malcolm Bennett, Samantha Bremner-Harrison, Elizabeth A. Chadwick, Frank Hailer, Stephen W. R. Harrison, Xavier Lambin, Matthew Loose, Fiona Mathews, Rachael Tarlinton

**Affiliations:** ^1^​ School of Veterinary Medicine and Science, University of Nottingham, Sutton Bonington, UK; ^2^​ Animal and Plant Health Agency, Addlestone, Surrey, UK; ^3^​ School of Biological Sciences, University of Aberdeen, Aberdeen, UK; ^4^​ National Wildlife Management Centre, Animal and Plant Health Agency, Sand Hutton, York, UK; ^5^​ School of Animal, Rural and Environmental Sciences, Nottingham Trent University, Southwell, UK; ^6^​ Vincent Wildlife Trust, Eastnor, Ledbury, UK; ^7^​ Organisms and Environment, School of Biosciences, Cardiff University, Cardiff, UK; ^8^​ School of Life Sciences, University of Nottingham, Nottingham, UK; ^9^​ School of Life Sciences, University of Sussex, Sussex, UK

**Keywords:** *Coronavirus*, mustelid, cricetid rodent, *Minacovirus*, stoat, SARS-CoV-2

## Abstract

Repeat spillover of severe acute respiratory syndrome coronavirus 2 (SARS-CoV-2) into new hosts has highlighted the critical role of cross-species transmission of coronaviruses and establishment of new reservoirs of virus in pandemic and epizootic spread of coronaviruses. Species particularly susceptible to SARS-CoV-2 spillover include Mustelidae (mink, ferrets and related animals), cricetid rodents (hamsters and related animals), felids (domestic cats and related animals) and white-tailed deer. These predispositions led us to screen British wildlife with sarbecovirus-specific quantitative PCR and pan coronavirus PCR assays for SARS-CoV-2 using samples collected during the human pandemic to establish if widespread spillover was occurring. Fourteen wildlife species (*n*=402) were tested, including: two red foxes (*Vulpes vulpes*), 101 badgers (*Meles meles*), two wild American mink (*Neogale vison*), 41 pine marten (*Martes martes*), two weasels (*Mustela nivalis*), seven stoats (*Mustela erminea*), 108 water voles (*Arvicola amphibius*), 39 bank voles (*Myodes glareolous*), 10 field voles (*Microtus agrestis*), 15 wood mice (*Apodemus sylvaticus*), one common shrew (*Sorex aranaeus*), two pygmy shrews (*Sorex minutus*), two hedgehogs (*Erinaceus europaeus*) and 75 Eurasian otters (*Lutra lutra*). No cases of SARS-CoV-2 were detected in any animals, but a novel minacovirus related to mink and ferret alphacoronaviruses was detected in stoats recently introduced to the Orkney Islands. This group of viruses is of interest due to pathogenicity in ferrets. The impact of this virus on the health of stoat populations remains to be established.

## Data Summary

The *Minacovirus* sequence assembled from this study has been deposited in the NCBI GenBank database under accession number OP933726. In addition, Illumina read datasets generated have been submitted under Bioproject accession number PRJNA897822, SRA accession numbers SAMN3158039, SAMN31580331 and SAMN31580344, and Biosample accession number SRS1567284850. Supplementary information for this paper is available at https://doi.org/10.6084/m9.figshare.24231709 [1].

## Introduction

Coronaviruses are a large and diverse group of enveloped RNA viruses found in diverse vertebrate hosts. Well-studied mammalian hosts, such as humans and domestic dogs, have multiple coronaviruses of several different subfamilies, although most of those of concern in mammals are members of the genera *Alphacoronavirus* and *Betacoronavirus* [2]. The severe acute respiratory syndrome coronavirus 2 (SARS-CoV-2) pandemic is thought to have arisen via a spillover from horseshoe bats (the natural hosts of the *Betacoronavirus* subgenus sarbecoviruses), with human infection probably via a ‘liaison host’ such as the raccoon dog (*Nyctereutes procyonoides*) or Malayan pangolin (*Manis javanica*) [3–5].

The overwhelming scale of the global human pandemic led to repeated spillovers and onward transmission into other mammalian species such as domestic cats (*Felis catus*) [6–8], farmed American mink (*Neogale vison*) [9–13] and Syrian hamsters (*Mesocricetus auratus*) [14, 15], and the establishment of a new reservoir in North American white-tailed deer (*Odocoileus virginianus*) [16, 17]. A wide range of other species are either able to be infected experimentally or have been subjects of sporadic case reports of SARS-CoV-2 infection or seroconversion including: cricetid rodents, felids, other small carnivores. mustelids, primates and bats (reviewed in [18, 19]).

SARS-CoV-2 infection in Muridae such as house mice (*Mus musculus*) and brown rats (*Rattus norvegicus*) may depend on the virus strain: initial studies with the original (Wuhan) strains of the virus failed to infect them [20, 21] and field studies failed to demonstrate evidence of infection in wild populations (27 *Mus musculus* and 97 *R. norvegicus*). Later variants did however cause infection in laboratory studies [21–24] and there have been several subsequent field reports of sporadic infection of rats [25–27].

The present study focused on species of wild animals present in Great Britain that were assessed to be of higher risk for SARS-CoV-2 spillover in 2021, when the study was begun. These included horseshoe bats (the subject of a separate report [28]), mustelids, small carnivores and cricetid rodents.

Thus far, no infection with SARS-CoV-2 has been reported in horseshoe bats (*Rhinolophus* spp.) in Britain or mainland Europe [28–30] although other related coronaviruses have been detected in these species. Reports in European deer prior to 2022 all failed to detect any exposure [31–33], but 57 % of fallow deer in Dublin, Ireland, seroconverted in early 2022 [34] and sporadic seropositivity in fallow and red deer in Spain in 2021–22 has also been reported [35]. Wild animal surveillance studies in mainland Europe have indicated sporadic detection in wild mustelids, including by quantitative (q)PCR in wild American mink (*N. vison*), particularly near farmed mink outbreaks, and one otter (*L. lutra*) [36–38]. Serological evidence of exposure has been described in 3/14 pine martens (*Martes martes*) and 2/10 badgers (*Meles meles*) [39], but other studies found no evidence of infection in 48 polecats (*Mustela putorius*), 163 badgers or cricetid and murid rodents (694 *Myodes glareolus*, two *Microtus arvalis*, 27 *Mus musculus*, 97 *R*. *norvegicus* and eight *Apodemus* species) [40–42].

## Methods

### Sample collection

A total of 402 samples from 14 wildlife species (Table 1) were collected through a network of wildlife researchers and volunteers engaged in wildlife conservation, monitoring or pest control. The majority of samples were collected during the human coronavirus disease 2019 (Covid-19) pandemic, with the exception of a small number of historical otter samples. The species targeted were primarily mustelids (otters *L. lutra*, badgers *Meles meles*, mink, pine martens *Martes martes*, weasels *Mustela nivalis*, stoats *Mustela erminea*) or cricetid rodents (water voles *Arvicola amphibius*, field voles *Microtus agrestis*, bank voles *Myodes glareolus*) with a small number of other species collected opportunistically (hedgehogs *Erinaceus europaeus*, common shrews *Sorex araneus*, pygmy shrews *Sorex minutus*, red foxes *Vulpes vulpes*, wood mice *Apodemus sylvaticus*). Ethical approval was granted by the University of Nottingham School of Veterinary Medicine and Science Committee for Animal Research and Ethics (CARE), and the University of Sussex Animal Welfare and Ethical Review Board.

**Table 1.** Species and sample type screened for SARS-CoV-2

Species	Oral swab	Rectal swab	Lung sample	Faecal sample	Total no. of animals	Sample collection dates
**Mustelids**						
Otter	–	–	75	–	75	Jan 2020–Apr 2022 (51 samples) Apr 2017–Dec 2019 (25 samples)
Badger	–	–	101	–	101	Apr 2021–Apr 2022
Mink	1	1	–	–	2	Aug 2021–Jun 2022
Pine marten	–	–	–	41	41	Mar 2020–Oct 2021
Weasel	–	–	2	–	2	Sep 2021
Stoat	7	7	–	–	7	Dec 2021
**Cricetid rodents**						
Water vole	–	–	–	108	108	Aug–Nov 2021
Bank vole	–	–	7	32	39	Jan–Feb 2022
Field vole	–	–	5	5	10	Nov 2021–Feb 2022
**Other species**						
Wood mouse	–	–	–	10	10	Jul 2021–Feb 2022
Common shrew	–	–	–	1	1	Jan 2022
Pygmy shrew	–	–	–	2	2	Jan 2022
Hedgehog	–	–	–	2	2	Nov 2022
Red fox	2	2	–	–	2	Apr 2022
**Total**					402	

Lung samples were taken from postmortem cadavers submitted to the Cardiff Otter Project (otters) or badgers found dead and submitted for tuberculosis monitoring to the University of Nottingham (badgers tested for covid were all confirmed culture-negative for *Mycobacterium tuberculosis* complex infections). Mink and stoat samples (oronasal and rectal swabs from cadavers) were provided from programmes for invasive species control and were from animals that were live trapped and euthanized (mink) or lethally trapped (stoats). Rodent samples were faecal samples from animals live trapped in Longworth or Elliot traps for population monitoring (faecal samples taken from traps or latrine/burrow sites), and from a small number of captive water voles from a licensed breeding and release programme. A small number of lung samples were taken from animals found dead. Weasel, hedgehog and shrew samples were from a small number of animals caught as bycatch in Longworth or Elliot traps. Pine marten samples were all faecal samples (scat) from environmental monitoring. Samples were collected from a variety of British locations (Supplementary Information, available in the online version of this article). Collectors were solicited by social media, notices in game and hunting organization newsletters, letters in the *Veterinary Record* and contact networks of wildlife organizations working with study participants and were provided with sampling packs including gloves, collection and shipping material and instructions. Samples were collected into RNAlater before either storage at −20 °C or direct shipping to The University of Nottingham, depending on the capacity of the collectors.

### RNA extraction, reverse transcriptase (RT) and RNA-dependent RNA polymerase (RDRP) gene coronaviruses generic conventional PCR and envelope gene sarbecovirus-specific real-time PCR

RNA extraction from lung tissue, faecal samples, rectal and oronasal swabs, and cell culture supernatant as a positive control was carried out using the Macherey-Nagel RNA tissue extraction kit as per the manufacturer’s instructions. The Wuhan SARS-CoV-2 strain positive control sample used throughout this study was kindly donated by Dr Christopher Coleman (Division of Infection, Immunity and Microbes, School of Life Sciences, University of Nottingham, UK). Reverse transcription was performed in two steps, using M-MLV-RT and random hexamer primers (Promega) as per the manufacturer’s instructions. All cDNA products were stored at −20 °C for conventional PCR.

A generic pan-coronavirus PCR assay [43] was used to amplify a 440 bp fragment of the coronavirus RDRP gene using Q5 Hot Start High-Fidelity DNA Polymerase (New England Biolabs cat. no.: M0493S). Primers were: F: GGTTGGGACTATCCTAAGTGTGA and R: CCATCATCAGATAGAATCATCATA. PCR products were purified using the Nucleospin extract II kit (Macherey-Nagel) according to the manufacturer’s instructions and were Sanger sequenced (Eurofins UK).

Real-time PCR was carried out using the Promega GoTaq Probe 1-Step RT-qPCR System (Promega) with Sarbecovirus-specific envelope gene primers as previously described [44]. Primers and probes were: F: ACAGGTACGTTAATAGTTAATAGCGT, Probe: FAM-ACACTAGCCATCCTTACTGCGCTTCG-BBQ and R: ATATTGCAGCAGTACGCACACA.

RNA and cDNA quality control was assessed via partial amplification of 108 bp of the beta actin gene using a published conventional PCR protocol [45]. Primers were F: CAGCACAATGAAGATCAAGATCATC and R: CGGACTCATCGTACTCCTGCTT

### High-throughput sequencing and genome analyses

RNA sequencing was performed on positive samples by Novogene, using the Illumina NovaSeq 6000 platform. Quality filtering and trimming to remove adapters, duplicates and low-quality reads was achieved using fastp v0.23.1 [46]. Kraken2 v2.1.2 was used for taxonomic classification of paired-end reads against the Kraken2 viral Refseq database [47] (retrieved 9 June 2022). Reads were assembled using the coronaSPAdes option in SPAdes genome assembler v3.15.4 [48] using default parameters. While CheckV v1.0.1, a fully automated command-line pipeline, was used for identification and quality assessment of contigs, contigs were also queried against the NCBI custom blastn (v2.12.0) viral database [49] (retrieved 3 July 2022).

Assembled contigs classified and assessed as complete alphacoronavirus genomes were indexed and extracted for downstream analysis using the SAMtools v1.16.1 faidx option [50]. Assembled genomes were annotated in Geneious Prime (v.2022.2.2) using NCBI coronavirus reference sequences for minacoviruses and tegacoviruses.

### Phylogenetic analysis

Complete coronavirus genomes, and extracted RDRP, spike and nucleocapsid nucleotide sequences from alphacoronavirus genomes assembled in this study, and a total of 22 reference alphacoronavirus genomes (all *Minacovirus* full genomes available and a selection of Refseq or well-characterized full-length isolates of tegacoviruses with the reference sequence of porcine epidemic diarrhoea virus, PEDV, as an outgroup, Supplementary Information) were downloaded from NCBI, and aligned using Mafft v7.490 [51]. Maximum likelihood phylogenetic trees were reconstructed based on complete coronavirus genomes, and four different genes using IQ-TREE v2.0.7 [52], with 1000 ultrafast bootstrap approximations using UFBoot2 within IQ-TREE v2.0.7 to evaluate branch support [53]. The ModelFinder function within IQ-TREE was used to select the best-fitting nucleotide substitution model for phylogenetic reconstruction [54]. Phylogenetic trees were visualized and annotated in FigTree v1.4.4 (https://github.com/rambaut/figtree/). Using the same approach trees were also similarly reconstructed for individual genes (25 spike and nucleocapsid genes as the S genes of this group of viruses are known to recombine) and for all available *Minacovirus* partial gene fragments of RDRP and spike (where there are a lot more sequences available than for other parts of the genome).

## Results

No animal sample tested positive on the *Sarbecovirus*-specific E gene qPCR.

Four (out of seven) stoat rectal swab samples (57 %) tested positive on the pancoronavirus PCR. Sanger sequencing of PCR products indicated that these were alphacoronaviruses of the *Minacovirus* group. No oral swab samples tested positive from the same animals. All stoat samples in this study were from the same population, and were sourced from the Orkney Islands stoat eradication programme (https://www.nature.scot/professional-advice/land-and-sea-management/managing-wildlife/orkney-native-wildlife-project) and are from the same recently introduced and heavily bottlenecked population. All except one sample in this study were from adult animals with a mix of males and females. Positive samples were from one adult male, one juvenile female and two adult females.

### Illumina sequencing taxonomic classification, and genome assembly

Taxonomic classification using Kraken2 identified reads assigned to other viral operational taxonomic units, but only reads classified to the *Coronaviridae* viral family are reported in this study. *De novo* assembly of datasets from the stoats yielded one full-length coronavirus contig of 28.1 kb (100 % quality, 99.9 % completeness) and two partial contigs (7.5 kb, 27 % quality, 26.6 % completeness; and 27.9 kb, 99 % quality and 99.1 % completeness) from a further two samples. The remaining sample did not yield any coronavirus contigs. The sequences had closest homology to alphacoronaviruses of the *Minacovirus* group. The complete full genome sequence of the most complete contig has been deposited in GenBank (accession number OP933726).

### Genome annotation and organization

**Fig. 1.**
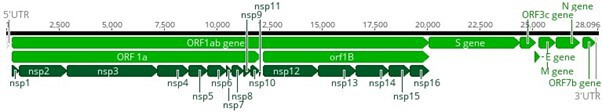
Genome organization of the stoat sequences derived from this study. ORFs are shown in light green, UTRs are shown in grey and regions with homology to non-structural proteins derived from feline coronavirus ORF1ab are marked in dark green.

Genome annotation demonstrated a typical *Alphacoronavirus* genome organization consisting of 5′ and 3′ UTRs, a large ORF1ab, encoding 16 non-structural peptides (nsp1-16) making up about two-thirds of the viral genome, and genes encoding four structural proteins: the spike (S), membrane (M), envelope (E) and nucleocapsid (N) (Fig. 1). Accessory proteins included ORFs with homology to ORF3c and 7b of feline coronavirus isolates. A number of smaller potential ORFs within these regions were also present, possibly corresponding to other accessory proteins with homology to the ORF3 or 7 of other *Minacovirus* and *Tegacovirus* isolates.

### Phylogenetic analysis

**Fig. 2.**
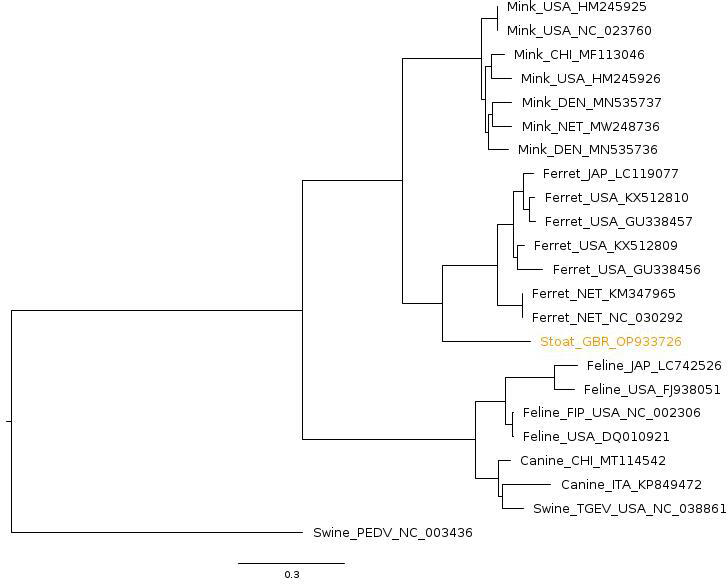
Maximum likelihood phylogenetic tree of full genomes of minacoviruses and tegacoviruses constructed with 1000 bootstrap approximation, rooted on the PEDV coronavirus reference sequence. Twenty-three full genomes were included. The sequence from this study is marked in orange. Sequences are named with species of origin, name of virus (if applicable) and a three-letter code for geographical origin (GBR=Great Britain, USA=United States of American, CHI=China, JAP=Japan, NET=Netherlands, DEN=Denmark) and GenBank ID. Bootstrap values are shown on nodes, the scale bar represents the number of substitutions per site.

Results from the maximum likelihood phylogenetic trees drawn using all available complete *Minacovirus* genomes and selected reference sequences for tegacoviruses demonstrated that the stoat sequence is most closely clustered with ferret isolates (Fig. 2). This relationship is consistent across analysis of individual genes (spike, nucleocapsid full genes) and in larger phylogenetic trees of all partial fragments of *Minacovirus* RDRP and spike available in the NCBI database (Supplementary Information).

## Discussion

This study found no evidence of widespread circulation of SARS-CoV-2 in our opportunistically gathered British wild carnivores and cricetid rodents in 2021–22. The study was designed to detect an incidence rate of 5 % based on our previous studies of alphacoronaviruses in British rodents [55] with a target of 73 samples per species calculated with the online Epitools sample size calculator [56]. For some species including otters and badgers, for which there were ongoing post-mortem studies of found dead (largely road kill) animals [57–60], this sample size target was readily achieved. This target was also achieved for water voles facilitated by a network of wildlife monitoring and captive breeding for re-introduction [61]. However, we were reliant on volunteer submitters for other species and did not achieve the target levels of fresh samples for hedgehogs, foxes, martens and other species. We were also restricted in the type of sample collected for most species as it was primarily non-invasive (faeces or post-mortem rectal and oronasal swabs) that could be collected by network members. There remains a possibility that our study did not detect low-level circulation of SARS-CoV-2 in the species tested if infection is spatially restricted, that we targeted the wrong tissue sample type or that our samples were too degraded to detect virus with RNA-based methods. This is inherent to the design of a study such as this one seeking to provide the basis for a preliminary assessment with scope to implement a spatially stratified study.

The detection of another coronavirus in this study, however, indicates that the methods used and sample preservation were adequate for viral detection, at least at high prevalence, even in small populations of samples. Our study is also in line with other European wildlife studies indicating absence of widespread SARS-CoV-2 circulation in wild small carnivores and rodents, including wild American mink [38–42, 62, 63]. Detection of SARS-CoV-2 in one Eurasian river otter in lung tissue and nasal swabs and detection of a Eurasian badger-specific coronavirus in lung tissue from similar post-mortem monitoring programmes indicates that lungs are an appropriate target tissue for coronavirus monitoring in those species [37, 42]. SARS-CoV-2 is also readily detected in faecal samples in laboratory studies of rodents and carnivore species as well as field studies of farmed mink [64–67], indicating faecal sample screening is also an appropriate sample for coronavirus monitoring. Faecal samples or rectal swabs are also among the easiest and most acceptable samples for volunteer submitters to collect safely, promoting their use in disease surveillance.

Serological testing for SARS-CoV-2 seroconversion would have been a useful addition to this study as PCR-based testing used in our study can only detect current viral nucleic acid shedding whereas serology can detect prior exposure to the virus and give a longer term picture of virus exposure. The limitations of volunteer sample submission meant that for many species blood, tissue or body fluids (such as pleural effusion) from which reasonable antibody recovery could be expected were unavailable. Commercial pan-species serology tests for SARS-CoV-2 are available and have been used in other studies of wildlife exposure to SARS-CoV-2 [31, 33, 34, 68] . However, these kits are not validated for all species and we do not know what the cross-reactivity is with some of the more divergent coronaviruses detected recently in European wildlife [42]. The results of these tests are usually confirmed with virus neutralization assays with false positives a common finding [39, 69]. These caveats make serology testing for an expected low case rate hard to interpret and this work remains for follow-up studies.

The stoat alphacoronavirus reported in this study is a novel finding. The stoats all came from an eradication programme on the Orkney Islands, an archipelago off the northeastern tip of mainland Great Britain. They are not native to the islands having been first found in 2010, and cause considerable negative impact on breeding bird populations [70]. Despite the small number of samples collected (seven) more than half of them (four) were PCR positive on rectal swabs, suggesting that the prevalence of this virus in this population is high. Wider studies of this population and mainland populations would be warranted to gauge the prevalence of the virus in the larger (and source) populations.

The novel virus we identified in Orkney stoats is a member of the *Minacovirus* subgroup of the genus *Alphacoronavirus,* and clusters closely to mink and ferret coronaviruses. The ferret and mink viruses have a well-described pathogenicity, causing diarrhoea and sometimes a systemic disease syndrome, similar to feline infectious peritonitis in cats [71–79]. Wider studies of the pathogenesis of this virus in stoats and the impact of this on the species would be warranted. There are multiple reports of the ferret viruses in this group recombining with each other [73, 75, 80] and multiple reports of farmed mink infected with SARS-CoV-2 also being co-infected with minacoviruses at a high prevalence rate [13, 81, 82]. While recombination between SARS-CoV-2 and minacoviruses has not been observed it is a possibility that warrants monitoring, particularly in farmed mink outbreaks.

Of interest, the tegacoviruses (the most closely related alphacoronavirus clade to the minacoviruses) are known for readily recombining and jumping host species, with canine, feline and porcine recombinants in this group reported in multiple separate events [83–88]. Canine coronaviruses have also been reported multiple times in multiple locations in people with respiratory disease [89–91], making this group of viruses of concern for recombination and cross-species transmission potential and warranting monitoring.

Sequences in the *Tegacovirus* group have also been reported from raccoon dogs [88, 92], suggesting that there could be cross-species transmission between raccoon dogs, domestic cats, dogs, pigs and people. This is of particular concern as raccoon dogs are known to be able to be infected with and transmit SARS-CoV-2 and are one of the main suspects for the origin of the SARS-CoV-2 outbreak in humans [3, 93, 94].

The *Minacovirus* sequences isolated to date are all associated with mustelids of the genus *Mustela*, subfamily Mustelinae (ferrets, mink, stoats). However, there are relatively few studies of coronaviruses in mustelids. That is beginning to be rectified with the publication of SARS-CoV-2 monitoring studies, with a possible *Gammacoronavirus* identified in Chinese ferret badgers (*Melogale moschata*) and, most recently, isolates of a possibly new genus, *Epsiloncoronavirus,* in Italian badgers [42, 95]. The potential host and geographical ranges of these viruses remain unknown.

Overall this study adds to a growing picture of a lack of widespread SARS-CoV-2 circulation in wild European mammals, other than fallow deer. However, a novel alpha coronavirus of the *Minacovirus* sub-genus has been reported in an island population of stoats, adding much needed information on alphacoronavirus diversity in mustelids. The wider disease impacts and epidemiology of this virus in this species is, however, unknown and requires further study.
